# Significance of indeterminate pulmonary nodules in resectable pancreatic adenocarcinoma—a review

**DOI:** 10.1007/s00423-020-02049-w

**Published:** 2021-01-03

**Authors:** Li Lian Kuan, Ashley R. Dennison, Giuseppe Garcea

**Affiliations:** 1 Department of Hepatobiliary and Pancreatic Surgery, University Hospitals of Leicester NHS Trust, Gwendolen Road, Leicester, LE5 4PW UK; 2 Discipline of Surgery, The University of Adelaide, The Queen Elizabeth Hospital, Adelaide, South Australia Australia

**Keywords:** Indeterminate pulmonary nodules, Pancreaticoduodenectomy, Pancreatic adenocarcinoma, Recurrence, Metastases

## Abstract

**Background:**

The clinical significance of indeterminate pulmonary nodules (IPN) in patients with resectable pancreatic adenocarcinoma (PDAC) is unknown. The rate of detection on IPN has risen due to enhanced staging investigations to determine resectability. IPNs detected on preoperative imaging represent a clinical dilemma and complicate decision-making. Currently, there are no recommendations on the management of IPN. This review provides a comprehensive overview of the current knowledge on the natural history of IPN detected among patients with resectable PDAC.

**Methods:**

A systematic review based on a search in Medline and Embase databases was performed. All clinical studies evaluating the significance of IPN in patients with resectable PDAC were included. PRISMA guidelines were followed.

**Results:**

Five studies met the inclusion criteria. The total patient population was 761. The prevalence of IPN reported ranged from 18 to 71%. The median follow-up duration was 17 months. The median overall survival was 19 months. Patients with pre-operative IPN which subsequently progressed to clinically recognizable pulmonary metastases, ranged from 1.5 to 16%. Four studies found that there was no significant difference in median overall survival in patients with or without IPNs.

**Conclusion:**

This is a first review on the significance of IPN in patients with resectable PDAC. The preoperative presence of IPN does not demonstrate an association with overall survival after surgery. The identification of IPN is a significant finding however it should not preclude patients with resectable PDAC from undergoing curative resection.

**Supplementary Information:**

The online version contains supplementary material available at 10.1007/s00423-020-02049-w.

## Introduction

The clinical significance of indeterminate pulmonary nodules (IPN) in patients with resectable pancreatic adenocarcinoma is unknown. The rate of detection on IPN has risen in the past decade due to staging investigations to determine resectability of primary tumour. Indeterminate pulmonary nodules detected on preoperative imaging represent a clinical dilemma and complicate decision-making.

 Indeterminate pulmonary nodule has been defined as an approximately rounded opacity more or less well-defined measuring ≤ 3 cm in diameter by the Fleischner Society glossary of terms for chest imaging and the British Thoracic Society [1, 2]. The detection rate of pulmonary nodules (< 8 mm) has increased significantly due to improvements in spatial resolution and broad availability of multidetector-row CT [3]. Characterization of small pulmonary nodule on CT images is very difficult because detailed morphologic features often cannot be perceived [4]. These findings are challenging to interpret, as IPN; non-calcified nodules can be detected in up to 25% of the general population [5, 6].

Pancreatic cancer is the 5th most common cause of cancer death in the UK, accounting for 6% of all cancer deaths (2017) [7]. Pancreatic cancer is expected to be the second or the third leading cause of cancer deaths in high-income countries by 2030 [8]. Pancreatic ductal adenocarcinoma (PDAC) and its variants are the most frequent type, representing 85–90% of all pancreatic neoplasms [9].

As most cases of pancreatic cancers present in their advanced stage, surgery is limited to those with resectable local disease without distant metastasis. Thus, it is vital that patients with newly diagnosed pancreatic cancer are accurately staged with proper protocols of computed tomography (CT) of the chest, abdomen and pelvis. The presence of an IPN may be an entirely benign entity or represent metastatic disease or (less likely) a primary lung malignancy which imaging has been unable to characterise. The uncertainty of IPN may expose patients to unnecessary investigations, delay or even preclude curative resection. The lung is the second most common site of metastasis (22–40%), after the liver for PDAC [10].

The aim of this review is to investigate the significance of IPN and its effect in patients with resectable PDAC. As well as to provide a comprehensive review of the current knowledge on the prevalence, and the natural history of IPN detected among patients with resectable PDAC.

## Methods and materials

A systematic literature search on Medline and Embase databases was undertaken. All clinical studies evaluating the significance of IPN in patients with resectable PDAC were included. Keywords used were indeterminate, lung nodules, pulmonary nodules, pancreatic ductal adenocarcinoma and pancreatic resection. The search duration performed was from January 2000 to March 2020. Due to paucity of data on this topic, the duration of web search was broadened to the last two decades. The search was restricted to English-language studies. PRISMA guidelines for systematic reviews were followed.

Eligibility criteria includes studies detailing outcomes of IPN detected in patients undergoing pancreatic resection for PDAC in adults over 18 years, the  presence of IPN evident on pre-operative imaging, patients undergoing surgical resection (pancreaticoduodenectomy/Whipple procedure, distal pancreatectomy, total pancreatectomy) for PDAC with curative intent.

Potentially relevant studies were identified by the title and abstract. Available full-text papers were obtained and assessed in detailed. A specifically designed data form was used to collect all relevant data. Data collection and analysis were carried out independently by two researchers. A third reviewer resolved any discrepancies found by the first two reviewers. The Newcastle-Ottawa Quality Assessment Scale was utilised for the assessment of the quality of studies.

The primary outcomes of the review were to identify the significance and natural history of IPN in patients with resectable PDAC.

## Results

**Fig. 1:**
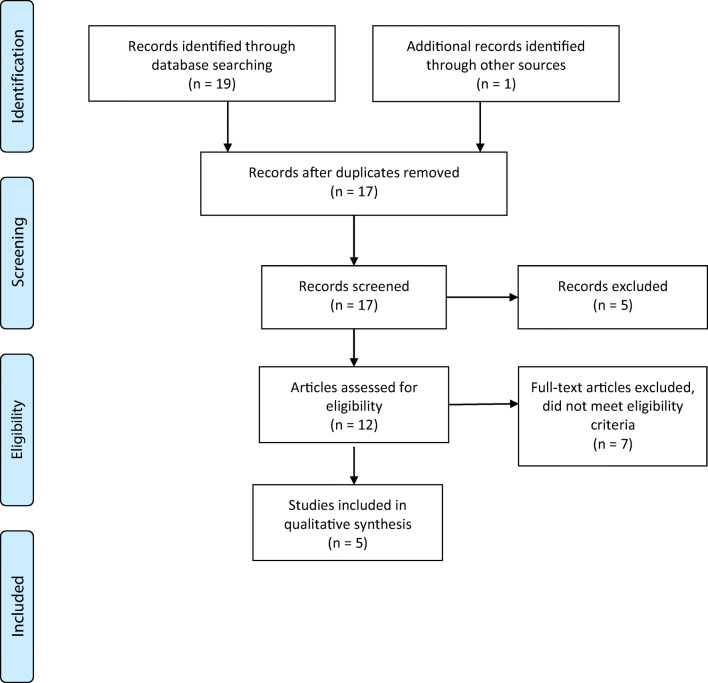
PRISMA diagram

The systematic search yielded nineteen items, which were screened by the title and abstract. Five studies met the predetermined eligibility criteria. The selection process of studies included in the review is outlined in the PRISMA diagram (Fig. 1). Five studies, with a total of 763 individuals with IPN who underwent pancreatic resection for PDAC were reviewed (Table 1). All studies were single institution, and retrospective cohort analyses. Due to the scarcity of available studies on this topic, a conference abstract paper was included in the review. The baseline characteristics of demographic and clinicopathological characteristics were similar among patients with and without IPN.

**Table 1:** Characteristics of studies examining the significance of indeterminate pulmonary nodules in patients undergoing resection for pancreatic ductal adenocarcinoma

Study	Title	Year	Study type	Location	Number of cases with CT scan	Patients with IPN	Mean age (years)	Gender
Chang et al. [11]	Natural History of Preoperative Subcentimeter Pulmonary Nodules in Patients With Resectable Pancreatic Adenocarcinoma	2014	Retrospective cohort, single institution	St Louis, MO, USA	329	59 (18%)	67	31 (53%) male 28(47%) female
Poruk et al. [12]	What is the Significance of Indeterminate Pulmonary Nodules in Patients Undergoing Resection for Pancreatic Adenocarcinoma?	2015	Retrospective cohort, single institution	Baltimore, USA	374 (16.2%)	183 (49%)	67.8	49% male 51% female
Mehtsun et al. [13]	Are Staging Computed Tomography (CT) Scans of the Chest Necessary in Pancreatic Adenocarcinoma?	2018	Retrospective cohort, single institution	Boston, USA	632	451 (71%)	66	205 (45%) male 246 (55%) female
Kazarian et al. [14] (conference abstract)	Clinical outcome of pancreatic cancer patients with indeterminate pulmonary nodules	2019	Retrospective cohort, single institution	Iowa, USA	1182 (total)*	50	68	N/A
**Wanjam et al. [15]	Resected pancreatic ductal adenocarcinomas with recurrence limited in lung have a significantly better prognosis than those with other recurrence patterns.	2015	Retrospective cohort, single institution	Baltimore, USA	209	20 (18 *post-resection—on surveillance CT)(2 pre-resection)	65.2 years (median)	54% male

*1182 pancreatic cancer patients from the Institutional Oncology Registry. Details on patients’ exclusion were not included

**Study focused on recurrence and looked retrospectively for IPN. Out of the 28 patients with lung recurrence, 24 patients had pre-operative lung nodules, of which 18 (75%) had IPN in their surveillance CT scans prior to the definitive diagnosis of lung recurrence

### Size of IPN

The cut-off size of IPN used by Chang et al. [11] and Mehtsun et al. [13] was under 1 cm; however, Poruk et al. [12] used a range of over 1 cm but less than 3 cm. The other studies did not report the definition of IPN used in their analysis. The median size of IPN ranged from 0.5 [11] to 0.65 mm [12].

### Number of IPN

A single study reported the median number of nodules as two, and observed six (17%) of IPNs that underwent enlargement [11]. The same study reported on the specific radiological characteristics of IPN; bilateral 4.3%, calcified 5.8%, solid 9.4% and spiculated 0.6% [11]. The prevalence of IPN was reported from a range of 18% [11] to 71% [13]. A comparison of the studies on IPN is shown in Table 2.

**Table 2:** Characteristics of indeterminate pulmonary nodules in patients undergoing resection for pancreatic ductal adenocarcinoma

Study	Definition of IPN	Number of IPN	Imaging modality	Size of IPN	Radiologic characteristics	Number of IPN that became malignant	Investigation/management of IPN post-operative
Chang et al. [11]	< 1 cm	N/A	CT or MRI (2 mm cuts through the upper abdomen and pancreas with a triple phase)	5 mm	Bilateral 14 (4.3%) Calcified 19 (5.8%) Solid 31 (9.4%) Spiculated 2 (0.6%)	5 (1.5%)	4 thoracentesis with cytology positive for malignant cells. 1 pulmonary metastases at autopsy.
Poruk et al. [12]	≥ 1 cm well-defined lung nodule(s) < 3 cm in diameter	One (*n* = 86, 46.9%) Two (*n* = 36, 19.6%)	CT	6 mm (range 0.2–2.7 cm) 1 ≤ cm (*n* = 159, 86%), five patients had nodules ≥ 2 cm but ≤ 3 cm	N/A	29 (16%)	10/29 (34%) had lung biopsy:malignant 1 had lung biopsy: benign 4 (14%) underwent resection of the lung nodule 16 (55%) had chemotherapy
Mehtsun et al. [13]	< 1 cm well-defined, non-calcified lung nodule(s)	N/A	CT	N/A	N/A	19 (4%) lung-only metastases 109 (24%) developed lung and abdominal metastases	6 (32%) underwent wedge pulmonary resection
Kazarian et al. [14] (conference abstract)	N/A	N/A	CT	N/A	N/A	32% lung-only metastases (total sum, not all may be from IPN)	N/A
Wanjam et al. [15]	N/A	N/A	CT	N/A	N/A	N/A	Not specified *67% of the patients received one or more anti-cancer treatments including surgical resection of oligometastases, chemotherapy and radiation therapy.

*Study focused on recurrence and looked retrospectively for IPN

### Progression of IPN to metastatic lung disease

Poruk et al. reported that 29 (16%) of patients with pre-op IPN subsequently progressed to have clinically recognizable metastatic lung disease at the location of the prior IPN based on radiological assessment [12]. Mehtsun et al. reported the total population of patients which had IPN (*n* = 451); 19 (4%) developed lung-only metastases, and 109 (24%) developed both lung and abdominal metastases, whereas of the 269 resected patients, 7.8% developed lung metastasis only [13]. Chang et al. reported the lowest incidence of lung metastases from IPN, 5 (1.5%) [11]. Kazarian et al. reported  that out of 50 patients with IPN, 37 (74%) developed local recurrence or distant metastases, and of these, 32% were lung metastases [14].

### Follow-up and recurrence

The median follow-up duration of all the studies was 17 months (range 13–20 months). The median overall survival was 19 months (range 16–23 months). Median survival was comparable among patients who did (15.6 months) or did not (18.0 months) have IPN (*p* = 0.66) [12]. Patients with lung-only recurrence had a median survival after recurrence of 17.9 months compared to 6.5 months for other single site recurrence or 4.3 months for multiple site recurrence [14]. All of the full-text studies found that there was no significant difference in median overall survival in patients with or without IPNs who had underwent pancreatic resections. Mehtsun et al. concluded the presence of IPN on index chest CT was not associated with an increased hazard of death (1.10 [0.89, 1.37], *p* = 0.42) [13]. Malignancy was associated with the number of IPN in one of the studies; the presence of more than IPN was associated with the development of lung metastasis (relative risk 1.58, 95% CI 1.03–2.4; *p* = 0.05) [12]. The outcomes of IPN are shown in Table 3.

**Table 3:** Outcomes of indeterminate pulmonary nodules in patients undergoing resection for pancreatic ductal adenocarcinoma

Study	TNM staging pancreatic adenocarcinoma	R0 resection	Median follow-up duration (months)	Median overall survival (OS) (months)	Outcomes	Impact on survival
Chang et al. [11]	Stage 1: 2 (3%) Stage 2: 57 (97%)	N/A	16.2	20.3	Only increasing age (67.1 vs 63.5 years; *p* = 0.005) was associated with the presence of subcentimeter pulmonary nodule (SCPN).There was no difference in OS between patients with or without preoperative SCPN (16.1 vs 19.1 months; *p* = 0.201).No radiographic criterion of SCPN (including number, size, laterality, calcification, or contour) was associated with OS.	No statistically significant differences in the overall survival between patients with and without IPN (*p* = 0.201)
Poruk et al. [12]	N/A	137 (75%)	17.7	15.6	The presence of > 1 IPN was associated with the development of lung metastasis (relative risk 1.58, 95% CI 1.03–2.4; *p* = 0.05).Lung metastasis was not associated with survival (*p* = 0.24).In a subgroup analysis of those with a preoperative IPN, there was no statistical difference in survival for those who went on to develop clinically recognizable lung metastases (*p* = 0.59).Median survival was comparable among patients with (15.6 months) and without IPN (18.0 months ,) (*p* = 0.66).	No significant differences in the overall survival between patients with and without IPN (*p* = 0.59)
Mehtsun et al. [13]	Stage 1 :12 (6%) Stage 2 : 134 (71%) 3 : 20 (10%)		12.9	16	No difference in median overall survival in patients without IPNs (16.4 months) vs those with IPN (14.8 months, *p* = 0.18).	No significant differences in the overall survival between patients with and without IPN (*p* = 0.18)
Kazarian et al. [14] (conference abstract)	Stage 1: 6 Stage 2: 44	82%	20	23	Patients with lung only recurrence tended to have superior OS relative to other single sites (HR 2.05, CI 0.66–6.33, *p* = 0.21) or multiple sites (HR 2.30, 0.75–7.50, *p* = 0.15).Only a portion of IPNs develop into true lung metastasis.	N/A
*Wangjam et al. [15]	Stage 2: 96.4%	46.4%	16	17.5 months (total population) 27.8 months (lung only recurrence)	Multivariable analysis suggested a delay in the diagnosis of lung nodules as lung recurrences was associated with a shorter recurrence to death (HR = 4.51, 95% CI = 1.27–6.1, *p* = 0.020). 75% of patients were found to have indeterminate lung nodules on their surveillance CT prior to the diagnosis of recurrence in lung. Lung recurrence was associated with the longest median survival time following recurrence among all recurrence patterns.	No

*Study focused on recurrence and looked retrospectively for IPN

## Discussion

Vast improvements in radiology have increased the discovery of IPN in patients with resectable PDAC although the clinical significance of these findings remains to be ascertained. Judicious pre-operative scanning of the chest leads to findings that are often confounded by the presence of IPN for which metastasis cannot be excluded. The present review found no statistically significant differences in the overall survival between patients with and without IPN [11–13]. This important finding suggests that the presence of IPN should not preclude patients with resectable PDAC from undergoing curative resection.

The National Institute for Health and Care Excellence (NICE) guidelines recommend staging CT scan that includes the chest, abdomen and pelvis, as well a fluorodeoxyglucose-positron emission tomography/CT (FDG-PET/CT) to patients with localised disease on CT who will be having cancer treatment [16]. The National Comprehensive Cancer Network (NCCN) guidelines also recommend staging chest CT over chest X-ray for initial evaluation of pulmonary metastases [17].

Currently, there are no recommendations on whether the presence of IPN in these patients should be managed by further investigations, by delaying or forsaking pancreatic resection or by proceeding with pancreatic resection followed by aggressive postoperative surveillance. Two studies in this review have challenged the limited diagnostic yield of chest CT in the initial staging workup in patients with resectable PDAC [11, 13]. Chang et al. commented that subcentimeter pulmonary nodules often elude further diagnostic testing by largely falling below the level of detection for PET-CT scans [11]. However, a study by Joo et al. that looked at the clinical significance of small (< 1 cm) IPN with little or no ^18^F-FDG uptake on PET-CT images of patients with nonthoracic malignancies concluded that > 19% of the cases turned out to be malignant [4]. A national Dutch study on the routine utilisation of chest CT in the diagnostic workup for PDAC (head) revealed clinically significant lesions in 10% of patients, with 4% of these being metastases [18]. At present, the published data available to determine the role of PET in the evaluation of IPNs are limited.

Overall, this review indicated that 1.5–16% of patients with IPN will ultimately progress to develop lung metastases [11, 12]. However, this does not appear to influence OS following pancreatic resection.

The discovery of IPN often creates a clinical dilemma, which may also alter disease management. Patients are put through further procedures, such as transthoracic aspiration/biopsy or bronchoscopy, but also more invasive procedures such as thoracic surgical biopsies, which adds to the patient’s distress and to the burden of costs. Percutaneous biopsy of lung nodules plays a critical role in obtaining pathologic proof of malignancy, guiding staging and planning treatment [19]. However, it is not without risks. In a large study population of 15,865 patients who underwent a transthoracic needle lung biopsy for a nodule found on CT scan, 15% suffered a pneumothorax of which 6% required a chest tube, and 1% had a haemorrhage [20]. In addition to that, lung nodule biopsy may be associated with sampling error from increased difficulty in localizing small lesions [21]. Lung nodule size of less than 10 to 15 mm decreases the accuracy of percutaneous lung biopsies [20, 21]. Tumour seeding of the lung, pleura or chest wall from percutaneous biopsy is rare, occurring in 0.01 to 0.06%. [22]

The detection of IPN did not lead to changes in the management of the primary tumour in these studies. Current diagnostic limitations preclude satisfactory risk stratification of these nodules, which can complicate treatment decisions regarding major oncologic surgeries with high morbidity and curative intent [11]. Accurate identification and characterization of IPN may not be necessary pre-operatively in patients with resectable PDAC.

This review also observed a favourable clinical course with a prolonged time from recurrence to death in patients who had subsequent lung metastasis as the first site of recurrence compared to patients who developed recurrent disease in other sites. Patients with metachronous pulmonary recurrence alone who developed pulmonary metastases as a site of first recurrence from PDAC have improved survival compared to those who develop metachronous recurrence in other sites or pulmonary metastases as a second or synchronous site of recurrence [12, 14, 23, 24]. Pancreatic cancer rarely leads to a solitary lung metastasis; usually, it leads to multiple metastases [25], and if this is the case, the finding of an isolated IPN further diminishes the risk of metastatic disease in patients with resectable tumour without the involvement of other sites. Liver metastasis is the most common site of distant reccurence [26, 27]. PDAC appears to have a distinct clinical course based on the site of recurrence in metastatic disease. These findings are indicated of the biologic heterogeneity of PDAC [27]. The studies found that there was no difference in survival between patients with or without IPN on preoperative CT and this may be attributed to the relatively low percentage of patients with an IPN that progressed to clinically recognizable pulmonary metastasis.

Emphasis is placed on the importance of surveillance post-resection because the progression of IPN can be detected early and a subgroup of patients could undergo treatment, which may be potentially curable. Of the patients who developed pulmonary metastases, a few were treated with pulmonary resection with intent to cure, which demonstrated a survival benefit [12, 13, 15]. In the first ever reported study, Arnaoutakis et al. showed that isolated lung metastases in PDAC can be considered for pulmonary resection with curative intent in a small number of selected patients [28]. It must be stressed, however, that this is not a widely accepted treatment for metastatic PDAC. 

This review attempted to identify prognostic factors that may predict the progression of IPN in respect of size, number and characteristics of nodules. Unfortunately, due to the limited data available, potential associations could not be appreciated. The Fleischner Society stated that the growth rate of lung nodule is the only cardinal parameter to indicate malignancy [3]. One volume-doubling time (VDT) corresponds to a 26% increase in nodule diameter, and this is the most sensitive marker used for growth rate estimation [29–31].

In view of IPN in other malignancies, as observed among patients undergoing resection for colorectal liver metastases, there were no significant differences between patients with and without IPN in respect of disease-free and overall survival [32]. Another study suggested that IPN does not significantly affect OS, but may predict earlier disease recurrence [33]. A systematic review on IPN at colorectal cancer staging concluded that that only 1 in 100 IPN proved to be metastatic disease and such a low risk suggests that IPNs should not cause further preoperative diagnostic workup or follow-up besides routine regimens [34]. A study on IPN in melanoma patients observed that baseline IPN are most likely benign, while interval IPN are high risk for metastasis, and the absence of volume increase of IPN within 6 months excluded metastasis in most patients [35].

This review has a few limitations. Heterogeneity among the studies and inconsistency in data reported preluded a meta-analytical assessment. Moreover, the detection rate of IPN reported in these studies may be lower than those in published reports as only patients with potentially curative PDAC were included. All the studies were from a single country (USA), retrospective, single-centred and consisted of a small sample size. A conference abstract was included in this review due to paucity of data. Despite the limitations, this review is important as it addresses the need for future research to establish guidelines with specific recommendations regarding IPN in patients with different primary malignancies.

## Conclusion

This is a first review conducted to study the significance of IPN in patients with resectable PDAC. True IPNs detected during staging of pancreatic cancer do not appear to have a negative impact on survival. The preoperative presence of IPN does not demonstrate an association with OS after surgery. The identification of IPN should not preclude patients with resectable PDAC from undergoing curative resection however, should be subjected to intensive surveillance post-resection.

## Supplementary information

ESM 1 (DOCX 179 kb).

## Data Availability

Provided upon request.
